# A four-year, systems-wide intervention promoting interprofessional collaboration

**DOI:** 10.1186/1472-6963-12-99

**Published:** 2012-04-20

**Authors:** Jeffrey Braithwaite, Mary Westbrook, Peter Nugus, David Greenfield, Joanne Travaglia, William Runciman, A Ruth Foxwell, Rosalie A Boyce, Timothy Devinney, Johanna Westbrook

**Affiliations:** 1 Faculty of Medicine, University of New South Wales, Kensington, NSW 2052, Australia; 2 The University of South Australia, GPO Box 2471, Adelaide, South Australia 5001, Australia; 3 University of Canberra, Canberra, ACT 2601, Australia; 4 University of Southern Queensland, Toowoomba, QLD 4350, Australia; 5 University of Technology, 15 Broadway, Ultimo, Sydney, NSW 2007, Australia

**Keywords:** Systems research, Action research, Intervention, Change, Interprofessionalism, Survey, Longitudinal research, Attitudinal improvement, Collaboration, Socio-ecological theory

## Abstract

**Background:**

A four-year action research study was conducted across the Australian Capital Territory health system to strengthen interprofessional collaboration (IPC) though multiple intervention activities.

**Methods:**

We developed 272 substantial IPC intervention activities involving 2,407 face-to-face encounters with health system personnel. Staff attitudes toward IPC were surveyed yearly using Heinemann et al's *Attitudes toward Health Care Teams *and Parsell and Bligh's *Readiness for Interprofessional Learning *scales (RIPLS). At study's end staff assessed whether project goals were achieved.

**Results:**

Of the improvement projects, 76 exhibited progress, and 57 made considerable gains in IPC. Educational workshops and feedback sessions were well received and stimulated interprofessional activities. Over time staff scores on Heinemann's Quality of Interprofessional Care subscale did not change significantly and scores on the Doctor Centrality subscale increased, contrary to predictions. Scores on the RIPLS subscales of Teamwork & Collaboration and Professional Identity did not alter. On average for the assessment items 33% of staff agreed that goals had been achieved, 10% disagreed, and 57% checked neutral. There was most agreement that the study had resulted in increased sharing of knowledge between professions and improved quality of patient care, and least agreement that between-professional rivalries had lessened and communication and trust between professions improved.

**Conclusions:**

Our longitudinal interventional study of IPC involving multiple activities supporting increased IPC achieved many project-specific goals. However, improvements in attitudes over time were not demonstrated and neutral assessments predominated, highlighting the difficulties faced by studies targeting change at the systems level and over extended periods.

## Background

Much health systems research is underpowered or cross-sectional. The field is dominated by small-scale studies, where research designs typically fail to incorporate longitudinal measurements. Studies of interprofessional learning (IPL) and interprofessional practice (IPP) (collectively, interprofessional collaboration (IPC)) are no exception. By professionalism we mean the expertise, challenges and autonomy that characterise practitioners' clinical work. Typically interprofessionalism is defined as two or more health care team members from differing professions working together productively, each making unique contributions to common goals. There is a prevalent view that encouraging interprofessionalism leads to enhancements in the ways healthcare professionals relate, interact and communicate, with resulting gains in quality of care and patient safety [1-3]. Demonstrating this causal chain has proven difficult, and showing controlled or sustained improvements in IPC has challenged researchers.

Some studies with randomised controlled designs have demonstrated IPC gains. Localised interprofessional rounds [4], interprofessional meetings [5,6] and externally facilitated interprofessional audits [7] can, depending on context, contribute to improvements in care (for a detailed review, see Zwarenstein et al. [8]). Building on this research, we deployed an action research team to the field to strengthen health professionals' IPC endeavours across the Australian Capital Territory's health system (ACT Health) over four years [9].

Rather than measure quality of care, which is notoriously difficult to achieve at the systems level [10,11], we designed interventional activities to make concerted efforts to stimulate increased interprofessionalism. We actively sought to work with staff, promoting communication, trust, joint problem solving, knowledge sharing and mutually constituted care, and discouraging rivalries and hierarchical modes of working. The research design, based on socio-ecological change theory, aimed to induce improvements by construing the health service as a socio-ecological system amenable to multiple intervention strategies [12,13]. This involved purposefully ranging across organisational hierarchical levels and divisional boundaries and silos, working with diverse participants and groups, focusing on cultural, interprofessional and process improvements. Socio-ecological theory eschews single method studies, instead proposing that the dynamic interconnections between behaviour and complex environments must be taken into account for change to occur [14,15]. It invites interventional researchers to take a multi-level, multi-pronged approach.

The aims of the present study were to investigate whether: 1) Initiatives, workshops and activities supported by the intervention achieved their goals, 2) Attitudes of health staff toward IPL and IPP became more favourable during the course of the study as measured at yearly intervals and 3) Health staff judged the intervention had achieved its goals.

## Methods

### Intervention projects and activities

Seven investigators with expertise in social psychology, medicine, patient safety, science, allied health, statistics, nursing and economics contributed to this study. Three experienced social scientists engaged as field researchers worked with an experienced project officer in the health system, led by a senior executive. Ethics committee approval for this project was given by the UNSW Social/Health Research Human Research Ethics Advisory Panel [approval number 09-10-006] and ACT Health Ethics Committee [approval number ET.3/07.2740].

Interventional initiatives emerged from each year's audit of the health system's interprofessional activities [16,17] and interactions and negotiations between the field researchers, ACT Health's project staff, and health professionals in the health system [18]. Examples of initiatives are presented in Table 1. A purpose-designed research protocol [9] and tool [16] were employed to guide research activities and gather data. An approach modelled on collaborative enquiry [19] stimulated reflection by health professionals and identified or helped initiate IPC projects. Workshops promoting IPC were designed and delivered during the life of the study. At a minimum annual feedback mechanisms were initiated. With some target groups, more frequent interactions encompassing personalised contacts and scheduled meetings with participants were invoked. This was an approach we labelled 'formative evaluation feedback loops' (FEFLs) [9].

**Table 1:** Examples of interventional activities

Interventional initiative	Focus	Activities
**Enhancing IPL-IPP of Continuum of Care for Allied Health Managers**	The project investigated how a senior management meeting could be improved to enhance IPC and clinical governance	• Development and piloting of questionnaire survey
		• Cross sectional census: survey
		• FEFL reflection exercise

**IPL and IPP in Palliative Care**	To strengthen staff wellbeing through examining and reflecting upon staff attitudes and their ongoing experience of IPC	• Development and piloting of survey
		• Longitudinal survey results fed back to participants
		• FEFLs with team

**Enhancing the interprofessional capacity of the Integrated Multi-agencies for Parents and Children Together (IMPACT) Program**	To enhance intra- and inter-agency IPC with the aim of improving client outcomes	• Development and piloting of survey
		• Longitudinal survey results
		• Key informant interviews, working out ways to collaborate more effectively
		• FEFLs with program staff

**IPL in primary health care to encourage active patient self-management of chronic disease (PSMCD)**	The project aimed to 1) identify strategies to strengthen PSMCD; 2) improve IPC across the health and community services sectors; and 3) support improvements in patient health literacy	• Development and piloting of questionnaire survey
		• Longitudinal survey results
		• Key informant interviews, with information fed back to participants
		• Consumer focus groups
		• FEFLs with program staff

**Evaluating the enactment of the Clinical Care Cultural Charter**	To assess progress in the enactment of a staff-defined organisational cultural charter	• Development and piloting of survey
		• Survey of staff
		• FEFL at staff development session

### Survey samples and procedure

ACT Health had 4,996 staff (full-time equivalent) in 2010, a slight increase from 4,869 at the beginning of the study, serving a population of almost 352,000. We surveyed staff in 2008, 2009 and 2010. Questionnaires were distributed at meetings to approximately 74% of clinical teams and units. Taking account of those not rostered at those times 30% of staff who had the opportunity to return the questionnaire in 2008 did so (n = 449), 35% in 2009 (n = 525) and 31% in 2010 (n = 471). Of staff completing the 2010 survey 16% indicated that they had completed both prior surveys, 8% that they completed the 2009 survey only, and 2% that they completed only the 2008 survey. Of staff answering the 2010 survey 39% had not been working for ACT Heath at the time of the first survey and only 52% had worked for ACT Health at the time the study commenced. As the surveys were completed anonymously it was not possible to compare an individual's responses across time. Overall, this implies that the majority of respondents completed the survey on only one occasion.

In the analyses, the respondent groups for each of the three years were treated as independent. The proportions of respondents from the different professions differed marginally across the years. The percentages of participants in 2008, 2009 and 2010 were medicine (8%, 5%, 9%), nursing (40%, 52%, 45%), allied health (36%, 33%, 35%), administration (12%, 5%, 7%), and other professions (4%, 4%, 4%). Thus compared to other years nurses were over-represented in 2009 and administrators in 2008. Similar proportions of males (17%) and females (83%) responded on all occasions. Average time since graduation for respondents across years was similar (15.6 years) as was time spent working in ACT Health (4.5 years).

### Questionnaire

The questionnaire administered on the three occasions included 1) The Attitudes toward Health Care Teams Scale [20], which consists of two reliable, validated subscales; Quality of Interprofessional Care and Physician Centrality and 2) The Readiness for Interprofessional Learning Scale (RIPLS) [21]. The factor analysis from which the RIPLS was derived revealed three factors - Teamwork & Collaboration, Professional Identity and Roles & Responsibilities - which formed the basis of three subscales (see Table 2). However some items loaded positively and others negatively on the Professional Identity factor leading some researchers to present results for both Positive and Negative Identity subscales [22]. Others have combined these scores as we did; e.g., Lauffs 2008 [23]. Specifically, we reverse scored items 10-12, which reflect a negative professional identity, and added them to positive items 13-16. As some researchers [24] had reported low internal consistency for two RIPLS subscales we checked respondents' α scores for the RIPLS subscales. These proved satisfactory for Teamwork & Collaboration (α = 0.87) and Professional Identity (α = 0.81) but not for the Roles and Responsibilities subscale (α = -0.17) which was consequently omitted from the analyses. The term 'health care student' used in some RIPLS items was replaced with 'health professionals' to make items more relevant to participants. A later revised version of the RIPLS for postgraduate health professionals, published after the present study was planned, similarly substituted with the term 'health care professionals' [25]. When factor analysed, the postgraduate version yielded Teamwork & Collaboration and Professional Identity factors but the inclusion of new items led to the Roles and Responsibilities subscale being replaced with a factor called Patient Centredness [25].

**Table 2:** Scales in survey questionnaire

**Attitudes toward Health Care Teams Scale** *Subscales*: Scores 6 (strongly agree) to 1 (strongly disagree) Higher scores indicate acceptance of doctors' higher authority in health care teams	**Quality of interprofessional care **(14 items) e.g. 'The interprofessional team approach makes the delivery of care more efficient' Higher scores indicate agreement with value of interprofessional care **Physician Centrality **(6 items) e.g. 'Doctors have the right to alter patient care plans developed by the interprofessional team'
**RIPLS (Readiness for Interprofessional Learning Scale)** *Subscales*: Scores 5 (strongly agree) to 1 (strongly disagree): Higher scores indicate non-rigid, non-tribal professional identity	**Teamwork & Collaboration **(9 items) e.g. 'Team-working skills are essential for health professionals from different professions to learn' Higher scores indicate approval of interprofessional teamwork and collaboration **Professional Identity **(7 items) e.g. 'Clinical problem-solving skills can only be learned with health professionals from my own profession' (reverse scored)

The 2010 questionnaire included 10 items assessing the achievement of the project goals (see Table 3 for items). Respondents were asked to assess whether these goals had been achieved using 5-point scales (ranging from 5 'strongly agree', through 3 'neutral' to 1 'strongly disagree'). The α score for the set of goal items = 0.97. The surveys also requested demographic information.

**Table 3:** Results of ANOVAs comparing staff attitudes in 2008, 2009 and 2010

Attitude scales	Mean scores for years			F	df	p	Duncan range test results*
					
	2008 (n = 449)	2009 (n = 525)	2010 (n = 471)				
***Attitudes toward Health Team subscales***							

**Quality of IP Care^#^**	4.69	4.72	4.68	0.61	2,1284	0.545	

**Doctor Centrality^#^**	3.17	3.08	3.29	9.43	2,1360	< 0.001	2009 2008 2010

***RIPLS subscales***							

**Teamwork & Collaboration^§^**	4.15	4.21	4.15	1.70	2,1357	0.184	

**Professional Identity^§^**	4.00	4.04	3.96	2.91	2,1364	0.055	

# 6 point scale; ^§ ^5 point scale; * Means of groups with common underlining do not differ significantly from each other but differ significantly from other groups e.g. Doctor Centrality scores for 2009 and 2008 do not differ significantly from each other but are significantly less than 2010 mean.

### Analysis

Interprofessional projects were stimulated by the study. They were logged and documented, and included in a database. The documentation was reviewed and content-analysed and the projects categorised. The scores on the two *Attitudes toward Health Care Teams *and two RIPLS subscales of the groups tested in the three years were compared by ANOVAs. When an F value was significant a Duncan range test was performed to identify which years differed significantly from others. The assessments of the success of 10 goals of the study made by respondents in 2010 were tabulated and a total assessment index calculated for each item by subtracting the percentage of the group who disagreed that a goal had been achieved from the percentage that agreed. For example, if 29% agreed and 8% disagreed that communication had improved the assessment index for this item would be 21%. Assessment indices were ranked from 1 (goal most achieved) to 10 (goal least achieved).

## Results

The interventions realised over the period 2007-2010 were the initiation or support of 101 substantial IPC improvement projects involving 573 health system participants, 108 feedback sessions (with 1,010 participants), 25 educational workshops (594 participants) and 38 other interventional activities (230 participants), attempting to build systems-wide momentum in support of increased IPC. The interventions stimulated are typified by the examples listed in Table 1. Intervention activities were conducted widely throughout the health system including in acute services, aged care, rehabilitation, mental health, community health and cancer services, involving some 2,407 substantial face-to-face encounters between research team staff and health professionals.

**Figure 1:**
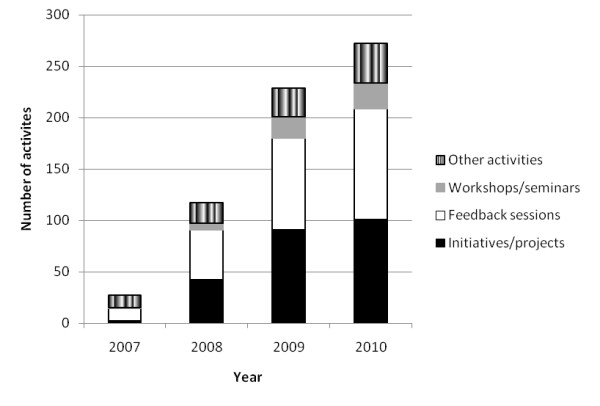
**Cumulative interventional activities**.

**Figure 2:**
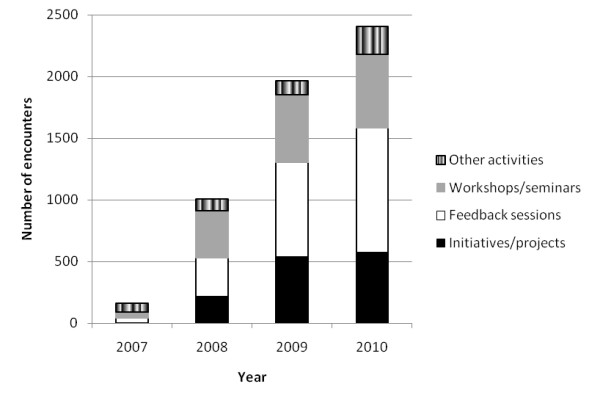
**Cumulative research staff face-to-face encounters with health system participants**.

The cumulative increases in logged projects, feedback sessions, workshops and seminars and other interventional activities are summarised in Figures 1 and 2. They show how the research team and project staff built momentum over time, interacting with many health professionals in different ways throughout the intervention. The accretion in activities demonstrates the action research methodology and the power of the FEFLs approach to engage health staff over time, in accordance with the first study aim.

**Figure 3:**
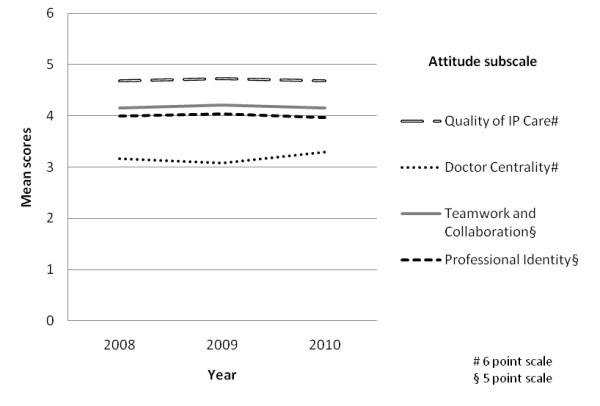
**Mean attitudes on four subscales, 2008, 2009 and 2010**.

Table 3 and Figure 3 present results relevant to the second study aim of enhancing attitudes toward IPC. The ANOVAs compare staff responses to the four attitude subscales. Attitudes regarding Quality of Interprofessional Care, Teamwork & Collaboration and Professional Identity did not change significantly over the three years. Scores increased significantly on the Doctor Centrality subscale in 2010 compared to the previous years during which they had remained similar. The Doctor Centrality scale measures whether respondents believe the physician's role is key and other professionals' role is to assist (p < .001). Respondents supported this notion more strongly at the end of the study, which was the reverse of what was predicted. Overall, there was no evidence that the study had enhanced attitudes toward IPL and IPP, and one contrary finding, that the intervention led to doctors being more central and not less.

The third study aim was to assess the success of the intervention. Table 4 shows that on average, for the 10 evaluation items, a third of respondents strongly agreed or agreed that the goals of the study had been achieved, with 10.5% disagreeing or strongly disagreeing. Over half of respondents (on average 56.9%) made a neutral response, neither agreeing nor disagreeing that a change had occurred. The assessment indices were used to determine the relative support for various goals. Those ranking most highly were: sharing of ideas between members of different professions, improved quality of patient care, and more positive attitudes toward teamwork. Least support was given for having achieved the goals of reduced between-professional rivalries and improved communication and trust between the professions.

**Table 4:** Assessment by the 2010 sample of achievement of study goals

Items: 'The IPL-IPP study has...'	% of staff responding			Assessment index	
	
	Strongly agree/ Agree	Neutral	Strongly disagree/disagree	(% agree - % disagree)	Rank*
**1. Improved communication between members of different professions**	30.8%	56.2%	13.0%	17.8%	9

**2. Improved trust between members of different professions**	29.8%	58.7%	11.4%	18.4%	8

**3. Helped individuals improve communication skills**	35.0%	55.6%	9.3%	25.7%	4

**4. Reduced between-professional rivalries**	20.8%	65.4%	13.8%	7.0%	10

**5. Resulted in better relationships between members of different professions**	31.4%	59.3%	9.4%	22.0%	6

**6. Resulted in more positive attitudes to team work**	37.0%	53.8%	9.1%	27.9%	3

**7. Led to sharing of knowledge/** **ideas between members of different professions**	39.4%	51.0%	9.6%	29.8%	1

**8. Led to more creative, integrated services**	31.6%	57.8%	10.7%	20.9%	7

**9. Improved clinical practice between members of different professions**	33.3%	57.2%	9.4%	23.9%	5

**10. Improved quality of care for patients**	37.3%	53.7%	9.0%	28.3%	2

**Average**	32.5%	56.9%	10.5%		

*Responses ranked from 1 most to 10 least achieved

## Discussion

Our concerted, longitudinal study, in partnership with a receptive health system which was prepared to engage in action research, produced or documented multiple activities promoting IPC. We stimulated many health system encounters with staff, and demonstrated substantial and growing participation in, and support levels for, interprofessionalism. However we found no evidence of improvements in attitudes over time, and in one case found that attitudes became less favourable; all despite the expertise provided by the intervention. This is consistent with Jha et al's conclusion that there is scant evidence of interventions influencing attitudes toward professionalism in medicine [26]. Nevertheless examination of the mean subscale scores indicates that, on the whole, staff possessed relatively favourable attitudes toward IPC from the very beginning, i.e., the time of the first survey (Figure 3). The mid-point of the Heinemann et al. subscales is 3.5. Mean scores on the Quality of Interprofessional Care subscale were substantially higher than this on all occasions and the means on the Doctor Centrality subscale were below the mid-point even when they rose at the end of the project. The mid-point of the RIPLS subscales is 3 and on the Teamwork & Collaboration and Professional Identity subscales the staff means were well above 3 each year. Thus the ACT Health workforce as a whole was well disposed toward IPC from the initial testing a year into the research. We do not know whether improvement occurred prior to the initial survey.

Notwithstanding much normative literature arguing in favour of IPC, according to these results, enabling greater levels of IPC across health systems over time is challenging. By expressing neutral views to the goal assessment questions the majority of staff did not commit themselves to judging either the success or failure of the project in achieving its objectives. The neutral response rate was high for this type of survey. Participants' reasons for checking neutral may have varied from feeling unable to make an assessment, holding mixed attitudes which were averaged to 'neutral', to lacking interest. If the wording of the items had specifically asked respondents to answer in terms of their own experiences they may have been less hesitant to make judgements. Nevertheless of staff making a definite assessment, three times more on average agreed goals had been achieved as disagreed that this was the case. The project was considered most successful in bringing about a sharing of ideas between members of the different professions, improving the quality of patient care and bringing about more positive attitudes toward team work. The least successful goals were the attempts to reduce between-professional rivalries, improve communication between the professions and improve inter-professional trust, which are core goals of IPC. Thus some of the goals of interprofessionalism appear to be more achievable than others.

A limitation of the study was staff turnover. This is a phenomenon all longitudinal health systems research faces. Many staff were not exposed to the full project so could the observed lack of change of attitudes over time be partly attributed to new staff? This possibility was not supported when we compared the 2010 attitude scores of staff who had only worked in ACT Health for two years with staff who had been employed for the full project. Newer staff expressed similar attitudes to their longer-term counterparts. Additionally, newer staff differed significantly from longer-term staff in their assessments of the project; on all items recent staff made fewer negative, more neutral and with one exception, more favourable judgements. Not surprisingly, longer exposure to the project was associated with greater likelihood of making a definite appraisal but that appraisal was more likely to be unfavourable. Perhaps we are detecting a form of world-weary jadedness in longer-standing staff, and more accommodating receptivity for IPC exhibited by newer, possibly younger recruits. The differing professional composition of the samples on the three testing occasions was also a limitation. However differences occurred primarily in the proportions of administrators and nurses. Examination of the professional groups' responses indicated that these two professions expressed more moderate attitudes on the attitude subscales while allied health and doctors held the more extreme attitudes, allied health expressing the most favourable and doctors the least favourable views about IPC.

Other study limitations include: lack of randomisation and a control group (although in mitigation, this was an attempt to induce systems-wide change rather than do a randomised controlled trial); weak capacity to identify causality between interventions and improvements; lack of baseline data; and incomplete knowledge of the contribution of different elements of the study toward influencing IPC. In research of this kind we cannot rule out the Hawthorne effect [27], a mechanism whereby any changed behaviour can be attributed to participants responding to being studied. However, the point of action research is to promote improvements. In any case, untangling Hawthorne from planned study effects is not possible with current research capacities.

## Conclusions

These findings highlight the difficulty faced by studies promoting change across entire health systems and over extended periods where staff are busy, have many existing routines, and encounter competing priorities. We have demonstrated that a study predicated on using multiple interventional strategies to involve differing hierarchical levels, applying a socio-ecological logic with multiple target groups to induce systems change in IPC, is feasible [28]. However, it may be that longer time periods are needed to show sustained gains in IPC gradients at the systems level. Studies of this kind must answer the question: how will we engage and maintain the interest of managers and staff in longitudinal action research projects sufficiently to make significant change? On average, of staff who did make a definite assessment over three-quarters believed that the goals had been met. However, primary strategies of the study centred on lessening interprofessional rivalry and distrust and these were judged as least successful. Accomplishing these seems to be particularly challenging [18,26,29]. Improving IPC in complex socio-ecological environments seems to be an entrenched [1], wicked [30] problem.

## Competing interests

The authors declare that they have no competing interests.

## Authors' contributions

JB is principal chief investigator on the project, conceptualised and led the research project and co-developed the first draft of the manuscript. MW conducted the quantitative data analysis and developed the first draft of the manuscript. PN contributed field work data and co-wrote the manuscript. DG contributed field data and co-wrote the manuscript. JT contributed field data and co-wrote the manuscript. BR is a chief investigator on the project, contributed clinical expertise and co-wrote the manuscript. ARF is a chief investigator on the project, contributed methodological expertise and co-wrote the manuscript. RAB is a chief investigator on the project, contributed clinical expertise and co-wrote the manuscript. TD is a chief investigator on the project, contributed organisational expertise and co-wrote the manuscript. JW is a chief investigator on the project, contributed methodological and statistical expertise and co-wrote the manuscript. All authors read and approved the final manuscript.

## Pre-publication history

The pre-publication history for this paper can be accessed here:

http://www.biomedcentral.com/1472-6963/12/99/prepub
